# Association of quality of nursing care with violence load, burnout, and listening climate

**DOI:** 10.1186/s13584-024-00601-3

**Published:** 2024-04-24

**Authors:** Sigal Shafran Tikva, Gillie Gabay, Or Shkoler, Ilya Kagan

**Affiliations:** 1 Jerusalem College of Technology; Head, Hadassah Research and Innovation Center in Nursing, Hadassah University Medical Center, Jerusalem, Israel; 2 School of Sciences, Multi-Disciplinary Studies, Achva Academic College, Arugot, Israel; 3 HEC Montreal, Montreal, QC Canada; 4 Nursing Department, Ashkelon Academic College, Ashkelon, Israel

**Keywords:** Burnout, Listening climate, Nurses, Patient, Quality of care, Violence load

## Abstract

**Background:**

Violence against nurses is common. Previous research has recommended further development of the measurement of violence against nurses and integration of the individual and ward-related factors that contribute to violence against hospital nurses. This study was designed to address these issues by investigating the associations between violence, the listening climate of hospital wards, professional burnout, and perceived quality of care. For this purpose, we used a new operationalization of the violence concept.

**Methods:**

We sought nurses to participate in the study through social media which yielded 765 nurses working in various healthcare systems across Israel who volunteered to complete a self-administered online questionnaire. 80% of the sample were hospital nurses, and 84.7% were female. The questionnaire included validated measures of burnout, listening climate, and quality of care. Instead of using the traditional binary measure of exposure to violence to capture the occurrence and comprehensive impact of violence, this study measured the incremental load of violence to which nurses are subjected.

**Results:**

There were significant correlations between violence load and perceived quality of care and between constructive and destructive listening climates and quality of care. Violence load contributed 14% to the variance of burnout and 13% to the variance of perceived quality of care. The ward listening climate moderated the relationship between burnout and quality of care.

**Conclusions:**

The results of this study highlight the impact of violence load among nurses and the ward listening climate on the development of burnout and on providing quality care. The findings call upon policymakers to monitor violence load and allocate resources to foster supportive work environments to enhance nurse well-being and improve patient care outcomes.

**Supplementary Information:**

The online version contains supplementary material available at 10.1186/s13584-024-00601-3.

## Introduction

Workplace violence is a global ‘epidemic’ that affects all healthcare professionals [1, 2]. Workplace violence includes incidents of threats, assault, and other offensive behaviors (including physical beating, kicking, slapping, stabbing, shooting, pushing, biting, and pinching), as well as incidents of psychological violence like rudeness, yelling, interrupting, bullying, undermining, and ignoring [3, 4]. Such violence has been recognized as an occupational hazard, and its negative consequences are well-known [5, 6]. By the early 1990s, the recognition of workplace violence toward nurses as an occupational risk in psychiatric settings was extended to other types of settings [7]. Since then, the great increase in research into workplace violence has contributed to raising awareness of the problem [8–10]. The COVID-19 pandemic has seen an increase in workplace violence with higher numbers of incidents of physical violence and verbal abuse and more difficulty in reporting incidents to management [10–13].

Workplace violence often increases the levels of distress, anxiety, depression, dissatisfaction with work, exhaustion, poor well-being, and other negative consequences for individuals [10, 14, 15]. On the organizational level, workplace violence is linked to higher turnover, lower morale, poor or missed nursing care, and increased burnout [16–18]. This is of great importance because nursing stands out as the profession with the highest levels of professional burnout [19–21]. This manifests as a progressive psychological response to chronic work stress with three main dimensions: (1) emotional exhaustion, (2) depersonalization, and (3) decline in professional efficacy [22, 23].

Consequences of burnout in nurses include poor physical health, diminished mental health, decreased self-compassion, work–home conflicts, decreased job satisfaction, and impaired work performance. Furthermore, there are associations between burnt-out nurses with higher numbers of medical errors, suboptimal patient care, and lower levels of work involvement – all of which have adverse effects on patients, threaten nurse retention, and increase hospital costs [24–27].

Workplace violence occurs within an organizational climate, which can moderate the condition in either direction [28]. Organizations with a pervasive safety climate are firmly committed to protecting patients and nurses from harm. This commonly involves promoting open, non-punitive communication regarding adverse events, and commitment to learning from such events to avoid their recurrence [29]. An organizational climate may also moderate the relationships between workplace violence and workers’ engagement [30]. More specifically, an organizational climate that emphasizes the quality of the provider-patient relationship and the quality of listening may mitigate workplace violence against nurses [31–34].

Listening has three components: (1) attention (to the speaker), (2) comprehension (of the speaker), and (3) (positive) intention (e.g., being empathic and non-judgmental) [35–37]. A *constructive* listening climate is present when one perceives the other person as paying attention to him/her, understanding him/her, and relating to him/her positively (non-judgmental, empathic, etc.). The dysfunctional opposite is defined as *destructive* [38]. Studies suggest that the ward’s climate of constructive listening may reduce nurses’ exposure to workplace violence [38, 39].

Classically, workplace violence has been viewed by researchers as part of a hospital’s quality dashboard, with hospital management recommended to examine trends in workplace violence incidents over time, evaluate the effects of workplace violence across units, and implement prevention programs [40]. However, monitoring workplace violence requires data concerning the magnitude of workplace violence across hospital units [40]. Previous studies on violence against nurses reduced it to a binary measure (i.e., exposed vs. not exposed). This binary approach fails to establish a measurement of the extent of exposure to workplace violence. Notably, a high prevalence of exposure in all areas invalidates comparisons across units [10]. This perspective ultimately monitor the continuum of workplace violence towards nurses: from no exposure at all to high exposure to workplace violence (i.e., more continuous properties). To address this issue, we have extended the measurement of violence by considering the exposure load of workplace violence on a continuum, namely, “Violence Load.”

**Fig. 1:**
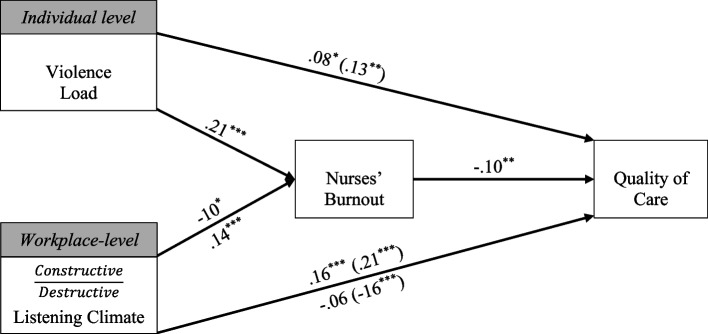
Path diagram with standardized regression coefficients (Beta). *Notes*. *p < .05, **p < .01, ***p < .001. Controlling for age, tenure, and gender. Coefficients in parenthesis are the direct (bivariate) association between variables. For Listening climate (as depicted in its rectangle): coefficients *above* the regression line reflect “Constructive” climate, while coefficients *below* the regression line reflect “Destructive” climate. The model boasts superior fit (Byrne, 2010): χ2(df) = 16.30 (2), *p* = .072; SRMR = .05; CFI = .96; NFI = .96; TLI = .92; GFI = .99; RMSEA (90% CI) = .08 [.05-.14], *p-close* = .058

 Although there has been extensive research focused on workplace violence towards nurses, burnout, and the effect on quality of care, very few studies have focused on the relevant factors at the organizational level. In line with the recommendations of eminent researchers, we studied both personal and context-related factors of workplace violence [41]. Thus, this study were to examine the (a) associations between violence load, burnout, and quality of care, b) associations between the ward’s listening climate, nurse’s burnout, and quality of care, and (c) the mediating effect of burnout on the relationships between ward’s listening climate and violence load to quality of care. The study model is shown in Fig. 1.

## Methods

### Sampling method

The minimum *a priori* sample size for the study, with a standard α error probability of 5%, power of 95%, and a fixed effect size of 0.15, was estimated by G*Power (v. 3.1.9.7) statistical software as *n* = 138 (and *n* = 204 for the effect size of 0.10). We therefore considered a sample size above 204 (as the stricter upper bound) as adequate for subsequent analysis.

A digital link to the anonymous online questionnaire was circulated among nurses via social network platform for specific nursing groups (Facebook and WhatsApp) from May to July 2020. We invited nurses to fill out the questionnaire with the following statement: “Staff nurses, please access a questionnaire that deals with violence towards nurses. We appreciate your time, and you can fill out the questionnaire using your preferred device.” This distribution method allowed us to reach out to nurses from different healthcare organizations and enabled the participants to respond anonymously to this sensitive topic. Before data collection, we conducted a pilot study among ten nurses to assess respondents’ understanding of the questionnaire.

Out of 1332 potential respondents who accessed the web link of the questionnaire, we excluded those with empty records and obtained a final sample of 765 nurses. Notably, this 58% response rate is above the response rate found in a meta-analysis on the adequacy of response rates in online surveys [42].

### Measures

The study survey examined (a) exposure to violence load, (b) the ward climate of listening, (c) burnout, (d) quality of care, and (e) socio-demographic characteristics. The measures used in the current research were translated from the original English into Hebrew by the back-translation procedure [43]. All measures had adequate psychometrics. Four experts, two senior researchers, and two senior nurses who study violence at work reviewed the instruments for relevance and clarity. They suggested a few changes in wording, which were accepted and incorporated into the final version of the survey. The full questionnaire can be supplied upon a reasonable request.

*Violence Load* was assessed by a scale of nine items relating to verbal and physical violence in the last 6 months [3]. The types of violence are verbal violence, verbal threats, destruction of property, minor physical violence, severe physical violence, use of a weapon or a sharp object, sexual harassment, and social shaming (Additional file 1). The respondents were asked to rate their experience of violence as: (1) never, (2) yes, exposed to *patient-perpetrated violence*. This study exclusively examined patient-perpetrated violence. In order to acquire continuity and relativity for this binary construct, the overall score was derived as follows: (A) When the participant replied “yes” once, they were given a score of 1 for violence ; (B) when the answer “never” was selected, the score given as 0; (C) the responses were summed to obtain an incremental increase in Violence Load and create a continuous variable with higher statistical variability, instead of a dichotomous response construct, such that higher scores represent higher violence load, and vice versa (i.e., higher scores reflect “higher load,” or occurrence/frequency, of violence). The final variable can be regarded as continuous, although a reliability coefficient could not be calculated.

*Quality of care* was assessed by six items previously used to measure the reported quality of nursing care in the context of abusive behaviors [38]. For example, “*In my ward, the treatment of patients who demonstrate violence behavior is incomplete*” or “*The level of care for violent patients, as compared to other patients in my ward, is low.”* Respondents were asked to rate each item on a Likert scale ranging from 1 (strongly disagree) to 7 (strongly agree). The overall score was represented by the mean. A higher mean score indicated a lower quality of care (a higher impairment of the quality of care) of sexually harassing patients.

Respondents were asked to rate each item on a scale from 1 (*strongly disagree*) to 7 (*strongly agree*). One item was recoded. The overall score was represented by the mean of the construct, with a higher score indicating a higher quality of care. Reliability (Cronbach’s Alpha Coefficient) was adequate, α = 0.78 [44].

*Burnout* was assessed using the 14-item Shirom-Melamed Burnout Measure [45]. Participants were asked to rank the statements on a scale from 1 (*never*) to 7 (*always*). A high mean score reflects high burnout. The reliability (Cronbach’s Alpha Coefficient) was high α = 0.9. For example, *“I feel physically fatigued”* and *I am too tired to think clearly.*

Ward’s climate of Listening to Patients was assessed using measurements of both perceived “constructive” and “destructive” listening [35, 36]. Respondents were asked to rate nine items (six items for constructive listening climate, and three destructive listening climate) on a scale from 1 (never) to 7 (always). Example items are: “*When nurses listen to patients, they listen carefully*” and “*When nurses listen to their patients, they try to understand what the patient is saying.”*

The overall score was the mean value of the totals. The reliability (Cronbach’s Alpha Coefficient) for constructive climate was high, with α = 0.91, and was adequate for destructive climate, with α = 0.75.

*Socio-demographic data* included gender, age, institution, specialty, place of birth, seniority, profession, form of employment, and position.

### Ethical considerations

The Institutional Review Board of Jerusalem College of Technology, the academic institution with which the first author is affiliated, granted ethical approval for this study (Approval #: 0313 − 17). The study adheres to Helsinki 1964 guidelines on ethics. Following the ethical approval, participants received a brief written explanation about the study’s aims. They were informed that the data collected would only be used for publication and statistical analysis. Completing the questionnaire served as consent to participate in the study.

### Data analysis

After demographic frequency analysis to test the research model (see Fig. 1), zero-order Pearson correlations were calculated to assess the baseline associations between the research variables.

Structural equation modeling was employed to assess the study model and the prevalence of common-method bias [46, 47]. Common method variance exists when the shared variance among variables is not due to the true underlying interrelationships but rather due to the measurement itself, namely self-reported data. Common method bias is a systematic error that can arise when respondents consistently rate items in a certain way, regardless of the actual relationships between the items [47]. Structural equation modeling was used because it allows researchers to model and analyze complex relationships among variables, handle both observed variables (measured variables) and latent variables (unobserved constructs), with more than one criterion, making it suitable for capturing intricate relationships that go beyond simple correlations [46, 48]. In addition, Structural equation modeling addresses measurement errors by allowing researchers to model the relationships between latent variables and their corresponding observed indicators enhancing the accuracy of the estimation of the tested relationships between variables in cross-sectional studies [46].

Two methodologies were employed to test the possible impact of common-method variance on the results [ 49,50]. These are (a) Harman’s single-factor method (all items are loaded on one common factor) and (b) a common latent factor method (all items are loaded on two types of factors – their expected factors and one latent common method factor). Analysis by Harman’s single-factor model accounts for only 21.81% of the explained variance and is a good fit [11, 46, 48–50]. Indices were: χ^2^(2696) = 8,491.17; *p* = .000; χ^2^/df = 3.15; Comparative Fit index = 0.78, Normed fit index = 0.75, The goodness of fit index = 0.86, SRMR = 0.13, and the root mean square error of approximation [90% CI] = 0.18 [0.14-0.29], *p-close* = 0.004. In contrast, the common latent factor model explained 20.37% of the explained variance: χ^2^(2583) = 6,741.63; *p* = .000; χ^2^/df = 2.61; Comparative fit index = 0.81, Normed Fit Index = 0.80, The goodness of fit index = 0.88, the difference between the observed correlation and the model implied correlation matrix was = 0.10, and the root mean square error of approximation [90% CI] = 0.11 [0.05-0.16], *p-close* = 0.017. Notably, these findings do not exclude the presence of common method bias [47]. However, as previously reported [47], we note that if the variance explained by the first emerging factor is less than 50% (*R* ^*2*^ < 0.50), then, in conjunction with a poor model fit for each analysis, common method bias is an improbable explanation of our findings (see also Table 1).

**Table 1:** Pearson zero-order correlation matrix and descriptive statistics (*n* = 765)

	Mean	SD	Gender	Age	Tenure	Violence Load	Destructive Listening	Constructive Listening	Nurse Burnout
Gender _(0 = men; 1 = women)_	1.85	0.36	-						
Age	41.48	9.97	0.08^*^	-					
Tenure	10.35	9.18	0.06	0.65^***^	-				
Violence Load	3.28	1.99	− 0.20^**^	0.07	0.07	-			
Destructive Listening	5.58	0.94	− 0.04	− 0.12^***^	− 0.09^*^	− 0.01	-		
Constructive Listening	2.00	0.94	0.05	0.12^***^	0.09^*^	0.01	− 0.58^***^	-	
Nurse Burnout	3.12	1.24	− 0.11^**^	− 0.16^***^	− 0.08^*^	0.16^***^	0.23^***^	− 0.21^***^	-
Quality of Care	3.47	1.27	− 0.02	0.14^***^	0.14^***^	0.13^***^	− 0.16^***^	0.20^***^	− 0.13^***^

Constructive/Destructive = Listening climate

*SD* Standard deviation

*p < .05, **p < .01, ***p < .001

Finally (Fig. 1), Structural equation modeling was also utilized [49] to test the mediation model, and full mediation analysis was employed with bootstrapping (95% bias-corrected confidence intervals and 5,000 re-samples; [46–50]. Bootstrapping can counteract any potential skew of the data from a normal distribution. In this case, the predictors are violence load and listening climate, while the mediator is nurses’ work burnout, and the criterion is the quality of care.

This study complied with STROBE guidelines [51].

## Results

### Participants

The research sample comprised 765 nurses, with females accounting for 84.7% of the sample. The persons in the sample ranged in age from 24 to 68 years (*M* = 41.48, *SD* = 9.97). Hospital nurses comprised 80% of the sample. Seniority in nursing ranged from 1 to 42 years (*M* = 10.35, *SD* = 9.18). Additional data are presented in Table 2. It is paramount to note that although most respondents were hospital nurses, we analyzed the data with and without the non-hospital participants. Since the changes in statistical results were negligible, we decided to keep the non-hospital nurses in the final sample to improve the power considerations and external validity.

**Table 2:** Demographic and background characteristics of the sample (*n* = 765)

Variable	Category	N	%
**Gender**	Female	642	84.7
	Male	116	15.3
**Organization**	General Hospital	421	56.8
	Community care	150	20.2
	Chronic hospital	170	22.9
**Organization type**	Public	634	85.1
	Private	50	6.7
	Both (combined)	61	8.2
**Professionally**	Licensed Practical Nurse (LPN)	15	2.0
	Registered Nurse (RN)	85	11.2
	Registered Nurse with BA	398	52.4
	Registered Nurse with MA or higher	262	34.5
**Religiosity**	Ultra-orthodox	9	1.2
	Religious	113	15.1
	Traditional	167	22.3
	Secular	423	56.4
	Atheist	38	5.1

There are a few missing values (i.e., participants who chose not to indicate or respond to a certain question) as can be seen that normal frequencies do not sum up to *N* = 765. However, the relative proportion (%) is accurate and accounts for missing values

### Correlational analysis

Table 1 presents the means and standard deviations of the study variables and the intervariable correlations. The modest strength of the correlations supports the notion that Common method bias is an improbable explanation for our findings.

### Mediating effects

Table 3 presents the findings from the path analysis to test mediation effects, while Table 4 reports the indirect (mediation) effect tests. Finally, Fig. 1 illustrates the findings on a path diagram.

**Table 3:** Path analysis results with standardized regression coefficients (beta)

Path			β	SE	t-test	Sig.
Violence Load	→	Burnout	0.21	0.02	4.15	< 0.001
Destructive Listening	→	Burnout	0.14	0.06	3.35	< 0.001
Constructive Listening	→	Burnout	− 0.10	0.06	-2.36	0.017
Burnout	→	Quality of Care	− 0.10	0.04	-2.63	0.008
Violence Load	→	Quality of Care	0.08	0.02	3.25	0.025
Destructive Listening	→	Quality of Care	− 0.06	0.06	-1.16	0.191
Constructive Listening	→	Quality of Care	0.16	0.06	3.56	< 0.001

Violence = exposure to violence. Constructive/Destructive = listening climate

**Table 4:** Indirect (mediation) effect analysis

Indirect effect’s Path					ε	SE	LL_95%_	UL_95%_	Sig.
Violence Load	→	Burnout	→	Quality of Care	− 0.03	0.10	-0.05	-0.01	0.010
*Constructive* listening	→	Burnout	→	Quality of Care	0.02	0.09	0.03	0.04	0.019
*Destructive* listening	→	Burnout	→	Quality of Care	− 0.02	0.10	-0.04	-0.01	0.009

Constructive/Destructive = listening climate. ε = standardized indirect effect size estimate. The number 95% = confidence level. LL = lower limit of 95% bootstrapping confidence interval (CI). UL = upper limit of 95% bootstrapping CI

The findings indicate that violence load contributes 14% to the variance of burnout and 13% to the variance of perceived quality of care. Table 4 reveals that three out of the four tested mediation effects are statistically significant: (1) burnout *partially* mediates the association between violence load and quality of care, (2) burnout *partially* mediates the association between a climate of constructive listening and quality of care, and (3) burnout *fully* mediates the association between destructive listening climate and quality of care.

## Discussion

This study extends the standard measurement of workplace violence to assess the cumulative effect of violence load on nurses. The study findings expand the existing knowledge on workplace violence in the field of nursing research with a focus on the impact of violence load, listening climates, and nurse burnout on quality of care. The results should motivate policymakers to employ this measurement in health systems and monitor and compare workplace violence data in and across units over time. Such monitoring will identify the units that most require intervention and the groups of nurses who are subjected to higher violence loads, thereby risking their personal wellbeing and jeopardizing quality of care.

Targeted strategies must be identified and implemented to ensure a safe and supportive work environment for nursing professionals. Monitoring violence load in and across units can identify better-performing units and allow them to be studied. Thus, they can potentially contribute to spread of effective interventions to reduce workplace violence.

Analyzing mediation pathways and identifying the relationships between constructive and destructive listening climates and nurse burnout also highlights the importance of fostering a positive work culture that promotes effective communication and support. Addressing destructive listening climates can also positively affect nurse burnout and the quality of care provided. Our findings are in accordance with those of studies in other industries, where a constructive listening climate significantly affected exposure to workplace violence [35, 36]. Notably, a byproduct of this study is the identification of a clear linkage between a constructive or destructive listening climate and the violence load. These effects may be explained by the emotional intelligence capability that constructive listening generates, which has been found to mitigate abusive behaviors [32]. In addition, our findings concerning listening climate support reports in the literature that meeting psychological needs help to reduce bullying and improve employee functioning [52, 53], while burnout mediates the effects of workplace violence on patient safety [54].

We recommend policymakers allocate resources for nursing management training programs designed to: (a) raise awareness of workplace violence among all nurses, encourage them to report workplace violence incidents and allocate time to discuss the nature, specific characteristics, and rates of occurrence over time; (b) establish policies that foster a supportive work environment, encourage open reporting of violence incidents, and prioritize nurse well-being; (c) allocate resources to ensure adequate staffing levels and support services to manage the impact of violence load on nurses; (d) adopt a trauma-informed care approach by integrating trauma-informed care principles into healthcare practices to address the psychological effects of violence load on nurses; and (e) support research into the consequences of violence load on nurse burnout and patient outcomes to guide evidence-based policymaking.

### Limitations

The use of single-source data and a cross-sectional design restricts causal inferences. We have tried to indicate possible causality by using Path Analysis. In addition, cultural influences may have impacted the results [55], and constructive listening may represent only one facet of a broader societal issue. In this study, we focused on the new measure of violence load on nurses, however, future research should explore the model in high workplace violence settings like emergency medicine, aged care, and mental health and replicate the studies in diverse countries to provide external and construct validity [56]. It may be informative to extend this approach to other health professionals in different healthcare settings since workplace violence extends beyond nursing and cultures [56]. It may also be useful to conduct longitudinal or cross-lagged studies designed to examine the association between violence load and post-traumatic stress [57].

## Conclusions

This study highlights the significance of the violence load and the ward’s listening climate as contributors to nurse burnout. The mediating role of burnout on the relationship between violence load, listening climate, and the quality of care provided by nurses underscores the importance of addressing workplace violence and promoting a supportive work environment in healthcare settings. Based on the findings of this study, we have developed a set of targeted strategies and policies, beginning with regular monitoring of violence load in units of health systems that would prioritize violence prevention and workplace support in order to improve nurse well-being and quality of care.

### Supplementary Information

**Supplementary Material 1.**

## Data Availability

The data supporting this study’s findings are available from the corresponding author [SST] upon reasonable request.

## Declarations

Anonymity was promised, and all data were coded without identifying details and were used for research purposes only. Completing the questionnaires was considered consent to participate in the study. All authors confirm that the protocol was performed in accordance with the ethical standards laid down in the Declaration of Helsinki and its later amendments or comparable ethical standards.

Authorship: We confirm that all authors meet the criteria of substantial contribution to all of the following: (1) the conception and design of the study, or acquisition of data, or analysis and interpretation of data, (2) drafting the article or revising it critically for important intellectual content, (3) final approval of the version to be submitted.

Reporting guidelines: STROBE reporting guidelines were used.
