# Correction: Association of quality of nursing care with violence load, burnout, and listening climate

**DOI:** 10.1186/s13584-024-00612-0

**Published:** 2024-08-09

**Authors:** Sigal Shafran Tikva, Gillie Gabay, Or Shkoler, Ilya Kagan

**Affiliations:** 1 Jerusalem College of Technology; Head, Hadassah Research and Innovation Center in Nursing, Hadassah University Medical Center, Jerusalem, Israel; 2 School of Sciences, Multi-Disciplinary Studies, Achva Academic College, Arugot, Israel; 3 HEC Montreal, Montreal, QC Canada; 4 Nursing Department, Ashkelon Academic College, Ashkelon, Israel

**Correction: Israel Journal of Health Policy Research (2024) 13:22**

10.1186/s13584-024-00601-3

The original publication of this article contained an incorrect author name. The incorrect and correct information is listed in this correction article, the original article has been updated.

Incorrect

Sigal S’hafran Tikva

Correct

Sigal Shafran Tikva

