# Supplementary material for: Association of quality of nursing care with violence load, burnout, and listening climate
Source: Isr J Health Policy Res. 2024 Apr 24;13:22. doi: 10.1186/s13584-024-00601-3 (PMC11040785; doi:10.1186/s13584-024-00601-3)
Supplement: Supplementary file 1 — Supplementary Material 1. [file 13584_2024_601_MOESM1_ESM.docx]

**Appendix 1.**

***Did you experience violent behaviors from patients in your department in the past six months? Please respond to every violent behavior in the list below.***

|  | **0**  **Never Experienced** | **1**  **Experienced** |
| --- | --- | --- |
| 1.**Verbal violence** (shouts, insults, curses - "stupid", "why who are you")  2.**Threatening verbal violence** ("at the end of the shift I will wait for you."  3. **Passive aggressive behavior** (threatening looks, muttering, intrusion into your physical space  4. **Vandalizing property as a protest** (throwing a chair, breaking a device, tearing up medical documents  5**."Mild" physical violence** (pushing, blocking a path)  6. **"Severe" physical violence** (punching, kicking, throwing objects at you)  7. **Threat with a sharp object or firearm**  8**. Shaming on social networks**  9. **Sexual harassment** |  |  |
